# Efficient post-acceleration of protons in helical coil targets driven by sub-ps laser pulses

**DOI:** 10.1038/s41598-017-06985-4

**Published:** 2017-09-07

**Authors:** H. Ahmed, S. Kar, G. Cantono, P. Hadjisolomou, A. Poye, D. Gwynne, C. L. S. Lewis, A. Macchi, K. Naughton, G. Nersisyan, V. Tikhonchuk, O. Willi, M. Borghesi

**Affiliations:** 1 Centre for Plasma Physics, School of Mathematics and Physics, Queen’s University of Belfast, BT7 1NN Belfast, UK; 2 Central Laser Facility, Rutherford Appleton Laboratory, Didcot, Oxfordshire OX11 OQX UK; 3 Department of Physics E. Fermi, University of Pisa, Largo B. Pontecorvo 3, 56127 Pisa, Italy; 4 University of Lyon, Ens de Lyon, Univ Claude Bernard, CNRS, Laboratoire de Physique, F-69342 Lyon, France; 5 National Institute of Optics, National Research Council (CNR/INO), A.Gozzini unit, 56124 Pisa, Italy; 6 Centre Laser Intenses et Applications, University of Bordeaux-CNRS-CEA, 33405 Talence cedex, France; 7 Institut für Laser-und Plasmaphysik, Heinrich-Heine-Universität, Düsseldorf, D-40225 Germany

## Abstract

The characteristics of laser driven proton beams can be efficiently controlled and optimised by employing a recently developed helical coil technique, which exploits the transient self-charging of solid targets irradiated by intense laser pulses. Here we demonstrate a well collimated (<1° divergence) and narrow bandwidth (~10% energy spread) proton beamlet of ~10^7^ particles at 10 ± 0.5 MeV obtained by irradiating helical coil targets with a few joules, sub-ps laser pulses at an intensity of ~2 × 10^19^ W cm^−2^. The experimental data are in good agreement with particle tracing simulations suggesting post-acceleration of protons inside the coil at a rate ~0.7 MeV/mm, which is comparable to the results obtained from a similar coil target irradiated by a fs class laser at an order of magnitude higher intensity, as reported in S. Kar *et al*., Nat. Commun, 7, 10792 (2016). The dynamics of hot electron escape from the laser irradiated target was studied numerically for these two irradiation regimes, which shows that the target self-charging can be optimised at a pulse duration of few hundreds of fs. This information is highly beneficial for maximising the post-acceleration gradient in future experiments.

## Introduction

Although characterised by exceptional transverse and longitudinal emittance, laser-driven ion beams (mainly produced through the target normal sheath acceleration mechanism (TNSA)^1^) present limitations in terms of their broad energy spectrum, large beam divergence and low particle numbers at high energies^2^. Control and optimisation of the beam parameters have therefore been an active research topic over the past decade in view of a wide range of potential applications, such as hadrontherapy^3–5^, warm dense matter^6, 7^, production of neutrons and radioactive isotopes^8–10^. In addition to addressing the divergence and broad spectrum of the beam, it is crucial, e.g. for medical applications^4^, to find stable and efficient ways of increasing the ion energy. Increasing the laser energy and intensity on target will naturally lead to an increase of the energy of TNSA-accelerated particles, although reaching hundreds of MeV energies may require large, multi-PW systems^11, 12^. Recently, a technique harnessing laser-driven EM pulses has been proposed^13^, which not only acts on the beam divergence and broad energy spectrum, but also provides a means of post-accelerating the ions. An accelerating gradient of ~0.5 MeV/mm was demonstrated using an university scale laser system, which is already well beyond what can be delivered by conventional RF accelerator technologies.

The technique for guided post-acceleration proposed by Kar *et al*.^13^ employs a target consisting of a helical coil (HC) attached to the rear of the interaction foil, so that the protons generated from the rear surface of the foil propagate through the coil, along its axis. As the ions are accelerated at the rear side of the foil, a high amplitude, ultra-short EM pulse is also launched into the coil, which propagates along the surface of the coiled wire with a velocity close to the speed of light^13–15^. The helical geometry allows a strong electric field region to exist inside the coil, where the radial and longitudinal components of the field act to collimate and accelerate the transiting protons. Since the EM pulse travels with a constant speed, the path for its propagation set by the coil geometry allows synchronisation of the travelling electric field region within the coil with a narrow slice of the proton beam spectrum, which can be tuned by varying the coil diameter and pitch. The simultaneous focussing, energy-selection and post-acceleration capabilities of the HC target therefore provide an attractive solution towards optimising laser-driven ion beams for the aforementioned applications.

Deploying the same technique at the TARANIS laser system at Queen’s University Belfast, UK, which is a 20 TW Nd-Glass laser system^16^, a highly collimated (<1° divergence cone) and narrow band (~10% energy spread) proton beam, containing >10^7^ particles at ~10 MeV was achieved by a uniformly pitched coil of ~5.5 mm length. Particle tracing simulations underpin the experimental data by showing the dynamics of beam focusing as well as post-acceleration/deceleration of protons (depending on the position of the protons relative to the travelling electric field region within the coil). An acceleration gradient of ~0.7 MeV/mm along the coil axis was estimated by the simulation from a comparison with the experimental data. This is higher than that obtained previously using the 200 TW fs laser system ARCTURUS^13^, delivering nearly an order of magnitude higher laser intensity than at TARANIS. The laser and target parameters were reasonably similar in both the experiments, except the pulse duration of the laser. Since the amplitude of the EM pulse is related to the target charging process following the laser interaction, the experiment implies that the charge accumulated on the target due to the laser irradiation has a weak dependence on the laser intensity. This observation is confirmed by comparison with the target charging simulation developed by Poyé *et al*.^17^. The process of charge accumulation at the laser-irradiated target takes a few ps, for a typical size of the laser irradiated foils, which is significantly longer than the laser pulse duration in both experiments. The simulations agree with the charge escape estimated in both short (ref. 13) and long-pulse (this paper) interaction regimes, and, by studying the dynamics of target charging for a given laser energy, indicate that charge accumulation in the target foil and EM pulse amplitude are optimized for pulse durations of a few hundred of fs.

## Results

**Figure 1:**
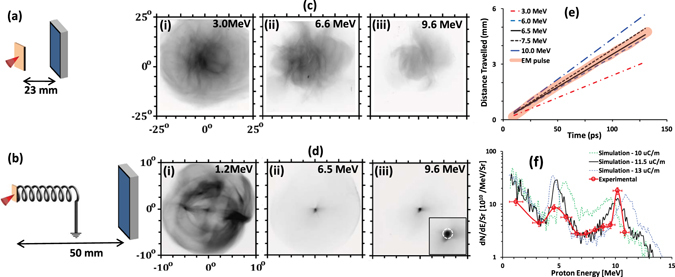
(**a**) and (**b**) show the schematics of the experimental setup, (**c**) and (**d**) show the proton beam spatial profiles obtained by the RCF stacks from flat foil and HC targets respectively. The spatial dimensions of the RCF images in (**c**) and (**d**) are shown in terms of the angle subtended with respect to the proton sources at the rear side of the foil. The insert in the (**d**-iii) shows a zoomed- in view of the focussed proton beam at the centre of the RCF image with the white circle showing the coil’s exit aperture. (**e**) Graph illustrating the synchronisation between the peak of the EM pulse travelling along the coil wire, projected on the coil axis (thick orange line), and the transit of different energy protons along the coil axis (thin lines as mentioned in the figure legend). (**f**) shows a comparison between on-axis proton beam spectra obtained in case of HC guided proton beam shown in (**d**), along with the proton spectra obtained from particle tracing simulation carried out for three different EM pulse amplitudes. The spectra were obtained by selecting a small area of 0.4 mm^2^ at the centre of the beam, corresponding to the exit aperture of the HC target.

Figure 1 highlights the salient features of the data obtained by employing the TARANIS laser^16^ at QUB (see methods section for more details about the experimental setup). In contrast to the typically observed divergent proton beams from flat foil targets, seen in Fig. 1(c), the HC target produced a tightly focussed beam of protons of energy around 10 MeV (see Fig. 1(d)). Since the protons would propagate ballistically to the detector after exiting the coil, the small spot size (less than the internal diameter of the coil) of the 10 MeV protons observed at ~50 mm from the target suggests a quasi-collimated (<1° divergence cone) beam being produced by the coil target. 1° represents an upper bound for the beam divergence considering the the diameter of the focused beam (~700 *μ*m) and the target to RCF distance (~50 mm). By selecting a small area of 0.4 mm^2^ at the centre of the beam, corresponding to the exit aperture of the HC target (as shown by the white dashed circle in the insert of Fig. 1(d-iii), across all the RCF layers in the stack, the proton spectrum along the coil axis was obtained (see the’Methods’ section for details about the process of spectral deconvolution from the dose deposited in the RCF layers). The on-axis proton spectrum obtained from this shot, as shown in Fig. 1(f), shows a narrow spectral peak of ~10% bandwidth at ~10 MeV, with a two orders of magnitude increase in proton flux compared to a flat foil reference shot. The number of protons within the spectral peak is estimated to exceed 10^7^.

Considering the speed (~0.96 of the speed of light in vacuum^13–15^) of the EM pulse travelling along the coil wire, the ~800 *μ*m diameter and ~320 *μ*m pitch of the coil would lead to the synchronisation of the EM pulse with ~7 MeV protons over the entire length of the coil. As shown in Fig. 1(e), 10 MeV protons from the laser irradiated foil would experience the EM pulse for less than 2 mm of the coil, which, as seen from the ~2 mm coil in ref. 14 is not enough to achieve the degree of collimation observed in Fig. 1(d). Therefore it is reasonable to assume that the focussed beam of 10 MeV protons was produced by post-acceleration of the ~7 MeV protons during their transit through the coil, due to their prolonged exposure to the longitudinal component of the electric field produced by the EM pulse. Assuming an EM pulse of ~15 ps FWHM with a nominal peak charge density of ~10 *μ*C/m (as measured at the TARANIS laser under similar interaction conditions^14^), a total charge of ~80 nC spread over 2 windings of the coil would produce a longitudinal electric field ~0.75 MV/mm, as can be estimated by using the simple charged-ring approximation discussed in ref. 13. Such intense field experienced over a length of ~5 mm would accelerate a proton by several MeV in energy, which is broadly consistent with the observed spectral peak at ~10 MeV.

In order to substantiate the experimental data, particle tracing simulations employing the code PTRACE^13, 18^ were carried out. Details about the simulation setup can be found in the Methods section. Using a broad, quasi-Maxwellian energy spectrum for the proton source, mimicking the spectral profile obtained from the reference flat foil target, simulations were carried out for the HC used in the experiment while varying the EM pulse amplitude (or the associated charge density). As shown in Fig. 1(f), the experimental spectrum was well reproduced by the simulation carried out using peak charge density of ~(11.5 ± 0.5) *μ*C/m, which is similar to what was measured previously by probing the EM pulse using the proton radiography technique^14, 15^. Simulations suggest that the two spectral peaks on either side of 7 MeV are produced due to the acceleration/deceleration of the leading/trailing part of the synchronised proton bunch within the coil, as previously discussed^13^.

**Figure 2:**
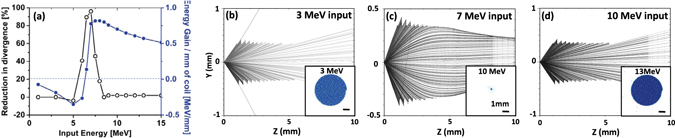
(**a**) shows the reduction in proton beam divergence and the gain in energy for different input proton energies as obtained from mono-energetic PTRACE simulations carried out for the case shown in Fig. 1(b)–(d) show 2D ray tracing of protons for three different input energies while transiting through the coil (0–5.5 mm) and beyond. The simulated proton beam spatial profiles at the detector are shown as inserts in the respective figures. The spatial scale in the insert corresponds to 1 mm at the detector plane.

In order to understand the dynamics of proton guiding and acceleration inside the coil, a series of simulations was performed using a divergent beam of mono-energetic protons at the source. As the protons emerged from the rear side of the coil, the beam divergence and energy gain were monitored, as plotted in Fig. 2(a). As can be seen, both the reduction in beam divergence and the energy gain are maximized at around 7 MeV input energy, which is in agreement with the estimated synchronisation window for the coil shown in Fig. 1(e). The longitudinal component of the electric field accelerates most effectively the leading part of the synchronised proton bunch. As shown in Fig. 2(a), the maximum rate of energy gain for protons of energies 7 MeV is ~0.7 MeV/mm, which is similar to what was estimated above using a simple charged-ring approximation. As shown in the insert of Fig. 2(b), the 7 MeV protons are accelerated to 10 MeV and produced a spot of diameter similar to the exit aperture of the coil (~700 *μ*m internal diameter), also in good agreement with the data obtained in the experiment.

## Discussion

A clear and noticeable result from these studies is the higher accelerating gradient of ~0.7 MeV/mm using the 20 TW TARANIS laser, when compared to the gradient of ~0.5 MeV/mm produced at the ARCTURUS laser delivering an order of magnitude higher intensity^13^. The strength of the EM pulse, which effectively carries a neutralising current from the target to ground, is related to the charge escape from the target following the interaction. However, the charging up of the target is a highly dynamic process, which self-consistently governed by the target potential as it evolves during the escape of hot electrons.

**Figure 3:**
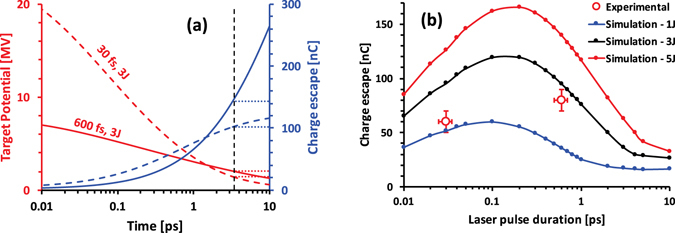
(**a**) Temporal evolution of target potential (red) and charge escape (blue) for 3 J, 600 fs (representative of the TARANIS system) - solid line, and 3 J, 30 fs (ARCTURUS system) - dashed line, obtained from the simple phenomenological model described in the text. A laser-to-electron conversion efficiency of 30% was assumed in both cases. The dashed black line indicates the time needed to spread the charge over a 2 mm diameter target disc After this time, target potential and escaped charge for 2mm-sized targets are assumed constant in the model, as indicated by the respective dotted lines. (**b**) shows the net charge escape from a 2 mm diameter target while varying the laser pulse duration, obtained from modelling of the target charging using the ChoCoLaT code^17^. The red data points show the total charge contained within the EM pulse measured at the ARCTURUS and TARANIS lasers with (*E*_*L*_, *τ*, *I*_*o*_) equal to (~1 J, ~30 fs, ~2 × 10^20^ W cm^−2^) and (~3 J, ~600 fs, ~2 × 10^19^ W cm^−2^) respectively.

A simple phenomenological model can be used at first to understand the dynamics as well as the relationship between the net charge escape and laser parameters, such as, for instance the pulse duration. The laser irradiated target acts as a dynamic capacitor, whose capacitance increases in time due to lateral spreading of hot electrons. As more electrons are allowed to escape, the target potential at a given time is determined by the electron spectrum, as only a finite number of electrons are available with kinetic energy above the target potential. Figure 3(a) shows the temporal evolution of the target potential and the net escaped charge from the target, while assuming a simple exponential electron spectrum, *dN*/*dE* = (*N*_0_/*U*_*p*_) *exp* (−*E*/*U*_*p*_), where *U*_*p*_ stands for the ponderomotive potential of incident laser. The target capacitance is defined as *C*_*T*_(*t*) = 8*ε*_0_(*r*_0_ + *ct*)^29^, where *ε*_0_ is the permittivity of vacuum and *r*_0_ the laser spot radius on the target. As can be seen from the graph, the target potential is very large immediately after the interaction. After this initial peak, the potential drops more rapidly for the higher intensity case. For a given laser energy and laser-to-electron conversion efficiency, which are broadly similar for the two experiments compared here, such behaviour is solely related to the electron spectrum produced during the interactions - the lower intensity interaction leads to a lower U_p_ but a higher N_0_. This effect would eventually lead, for an infinitely large laser irradiated foil, to a much higher number of escaping electrons for the low intensity case, as shown in Fig. 3(a). For a finite size of the target, however, the charging up reaches a saturation depending on the target’s self-capacitance, as shown by the dotted lines in Fig. 3(a) for a 2 mm diameter disc - the typical foil size used in the HC coils.

In order to simulate the net charge accumulation in the target, a self-consistent Coulomb barrier model, ChoCoLaT^17^, was employed. The height of the potential barrier in the model is defined by a competition between the hot electron ejection and the current of cold electrons compensating the positive charge building around the focal spot. The duration of the charging process depends on the cooling time of the hot electrons (typically 100 s keV/ps) and the lateral size of the target. A more realistic Maxwell-Jüttner distribution for hot electrons was assumed at the beginning of the simulation, with the effective temperature equal to the laser ponderomotive potential and 30% laser-electron conversion efficiency^19–21^. Because of the finite size of the target (2 mm), the charging process saturates at ~3.5 ps and the values for net charge escaped from the target for both TARANIS (pulse energy enclosed in the focal spot (*E*_*L*_) ~3 J, FWHM pulse duration (*τ*) ~600 fs and peak intensity on target (*I*_*o*_) ~2 × 10^19^ W cm^−2^) and ARCTURUS (*E*_*L*_ ~ 1 J, *τ* ~ 30 fs and *I*_*o*_ ~ 2 × 10^20^ W cm^−2^) laser parameters are found to be in good agreement with the experimental data, as shown in Fig. 3(b). As expected, the simulations suggest that a fewer number of higher energy electrons at a lower laser intensity is compensated by a commensurate increase of the number of low energy electrons, leading to a weak dependence of the net charge escape with the laser pulse duration for a given laser energy, i.e. with the laser intensity.

Increasing the laser energy simply leads to a commensurate increase in charge accumulation. However, the effect of laser pulse duration (assuming the same energy and focusing conditions) on the net escaped charge is more complex due to factors, such as the interplay between the hot electron temperature and population, as discussed above, and their cooling time. The hot electron collisional cooling can lead to a significant loss in electron mean energy over several ps of the charging period and plays an important role for long pulse durations (≳ps), as suggested by the simulation. Noticeably, the graph for net charge accumulation with laser pulse duration, shown in Fig. 3(b), has a maximum, which grows sharper with higher energy. For instance, the optimum pulse duration for our typical foil size is found to be ~200 fs, which can be easily attained by tuning the stretcher or compressor settings in a CPA system^22^. Maximising the charge accumulation on the target will maximise the EM pulse amplitude in an experiment, which would be beneficial in optimising the post-acceleration of ions by a helical coil.

## Conclusion

A quasi-collimated beam of 10 ± 0.5 MeV protons, containing ~2 × 10^7^ protons/MeV, was produced at the 20 TW TARANIS laser at QUB, by irradiating the HC target^13^ with a moderate laser intensity of ~2 × 10^19^ W cm^−2^. Simulations suggest that a linear accelerating gradient of ~0.7 MeV/mm was achieved within the coil, which is noticeably higher than the gradient produced by an irradiation with a order of magnitude higher intensity. The influence of laser intensity on the target charging was found to be not very effective, as long as the total energy conversion into hot electrons remains the same, which was explained by a detailed study of charging-up dynamics following laser interaction. The numerical models suggests that the net charge escape, hence the amplitude of the EM pulse launched into the HC coil, can be optimised with laser pulses of a few 100 s of fs duration. This information would be useful, for instance, in a multi-stage HC scheme^13^, to optimise the follow-on acceleration stages by tuning the laser pulse duration, while the highest intensity would be preferrable for the first stage to produce high energy seed protons from the TNSA mechanism.

## Methods

### Experiment

The experiment was performed at the TARANIS laser^16^ at Queen’s University Belfast, UK, employing CPA pulses of duration ~600 fs with energy ~7 J after the compressor. The short pulse was focused on the target by using a f/3 off axis parabola down to a spot of ~6 μm FWHM diameter, with ~40% of laser energy enclosed within the focal spot, delivering peak intensity ~2 × 10^19^ W cm^−2^. A multilayer stack of radio-chromic films (RCFs) was used to diagnose the proton beam’s spatial and spectral profiles. The RCF dose response was absolutely calibrated using a batch of the same type of RCFs exposed to different known proton doses obtained at a conventional particle accelerator^23^. Proton spectra were obtained by spectral deconvolution of the dose deposited in the RCF layers^24, 25^, by using an iterative algorithm which calculates the spectrum yielding the closest fit to the observed dose, similar to the procedures used in refs 23, 26, 27. Starting from the last RCF layer in the stack, the final spectrum is constructed sequentially by matching the observed dose in each RCF layers, while considering the energy response of the RCF layers (simulated by SRIM^28^) in the stack and subtracting the dose contribution in a layer by the protons stopping deeper in the stack. Two types of targets were used in the campaign as shown in Fig. 1(a): flat foils of 10 *μ*m Au in order to measure the proton beams produced by the TNSA mechanism, (b) helical coils attached to the rear side of 10 mm thick Au foils to study the effect of the coil on the transiting protons through the coil. The helical coil was made of aluminium wire of ~80 *μ*m diameter. The internal radius, pitch and length of the helical coil were ~350 *μ*m, ~320 *μ*m and ~5.5 mm respectively.

### Simulations

The particle-tracing simulations were carried out by using the code PTRACE^13, 18^, in which the charged particle is traced by solving equation of motion using a Runge Kutta fourth-order algorithm coupled with an adaptive step size monitoring routine. The helical coil was modelled in the PTRACE using a cylindrical coordinate system, and the electric field at any given point, and at a given time, was computed numerically by adding electric field vectors induced by every small element of the loop, while considering the position of the EM pulse in the wire at the given time. The dimensions of the HC were taken from the target images recorded prior to the shot. An EM pulse of 7 ps rise and 15 ps decay was used in the simulation travelling with a speed of 0.96c along the coiled wire, which was measured in previous TARANIS experiments (reported in refs 14, 15) with similar laser and target parameters. The proton source was located at the centre of the entrance plane of the coil, emitting protons towards the coil with a given energy spectrum and divergence. After the transit of the protons through the field region defined by the coil, the stack detector registers the location of every incident proton. The energy deposited in each layer of the stack is computed by using the stopping range of protons in the RCF plastic calculated by SRIM^28^ code. The description of the code ChoCoLaT used for simulating the target charging can be found in ref. 17.
